# A modified endoscopic full-thickness resection technique: double traction-assisted resection

**DOI:** 10.1055/a-2277-0540

**Published:** 2024-03-14

**Authors:** Yanjun Song, Xin Sun, Chang Ge, Ruyuan Li

**Affiliations:** 1 91623 Gastroenterology, Shandong University Qilu Hospital, Qingdao, China

With the extensive use of gastrointestinal endoscopy and endoscopic ultrasonography (EUS), the diagnosis rate of gastric submucosal tumors (SMTs) has increased. While most SMTs are thought to be benign, up to 13% have malignant potential, especially those originating in the muscularis propria layer 1 . With the advance of endoscopic technology, endoscopic treatment for SMTs has proven to be an effective, safe, economical, and most importantly, minimally invasive method for both accurate histopathological evaluation and curative treatment. With advances in endoscopic techniques, exposed endoscopic full-thickness resection (EFTR) has been demonstrated to be an effective and minimally invasive method for gastric SMTs originating from the deep muscularis propria layer or those with extraluminal growth tumor 2 . It is however a time-consuming procedure and not easy to perform, particularly for some lesions >2.0 cm. We here report a modified exposed EFTR technique, in which we performed EFTR assisted by double floss traction ( Video 1 ).

A modified endoscopic full-thickness resection technique, using double traction assistance, is used to resect a submucosal gastric tumor.Video 1

In this modified procedure, the first clip with floss is anchored to the resected part of the tumor surface to provide traction to expose the tumor. The second clip with floss is anchored to the submucosal tissues near the tumor to retract it into the gastric cavity and expose the serosal face of the tumor. First the margin of the lesion was marked using a DualKnife ( Fig. 1 **a** ), then sufficient submucosal injection was performed around the marks. Next, a C-shaped submucosal incision was made and submucosal dissection was performed to expose the tumor. A clip with floss was then fixed to the surface of the lesion to expose the tumor more clearly ( Fig. 1 **b** ). After further dissection of the tumor, a second clip with floss was anchored to the submucosal tissues near the tumor to retract it into the gastric cavity and expose the serosal face of the tumor ( Fig. 1 **c** ). Full-thickness resection was performed to resect the lesion and clips were used to close the defect ( Fig. 1 **d** ). The size of the tumor was about 3.0 × 3.5 cm ( Fig. 2 ), with pathology showing a low grade stromal tumor.

**Fig. 1:**
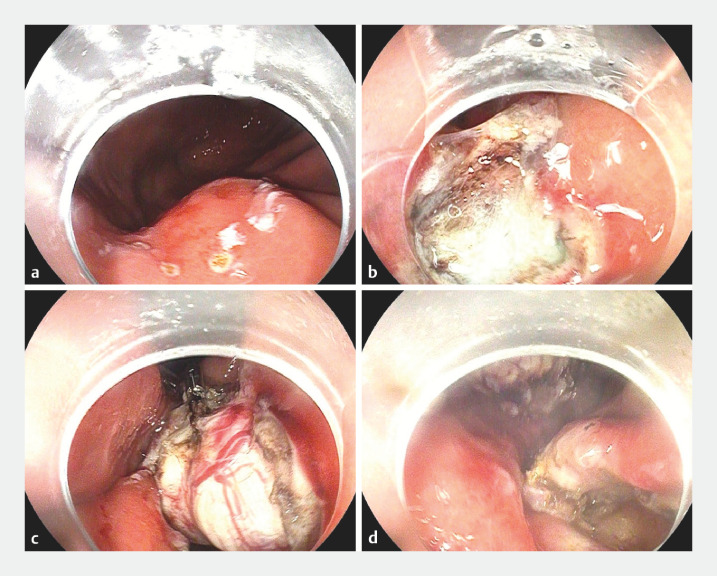
Endoscopic views showing: **a** the margin of the lesion marked using a DualKnife; **b** a clip with floss fixed to the surface of the lesion to expose the tumor more clearly; **c** a second clip with floss anchored to the submucosal tissues near the tumor to retract it into the gastric cavity, thereby exposing the serosal face of the tumor; **d** the appearance after full-thickness resection of the lesion.

**Fig. 2:**
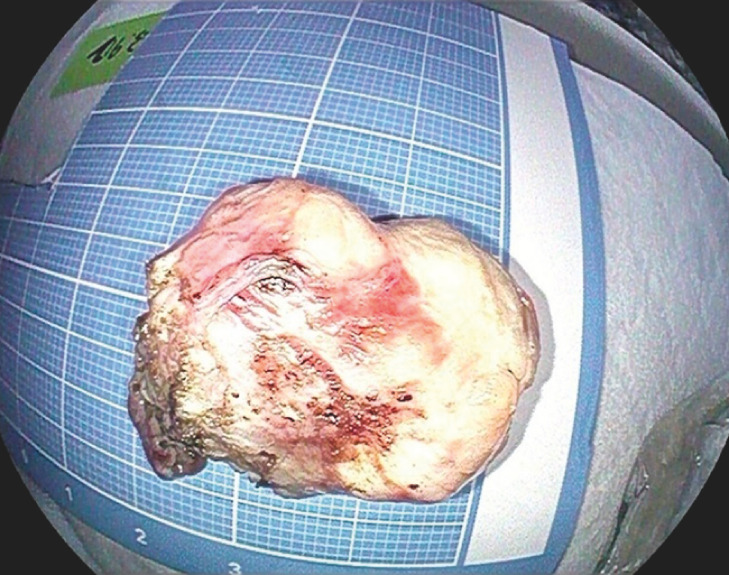
Macroscopic appearance of the tumor, which was about 3.0 × 3.5 cm in size.

This study is the first to propose a modified EFTR technique that is able to expose not only the surface of the tumor but also the serosal face of the tumor using a double traction-assisted method. We believe this technique is worthy of clinical promotion, although prospective studies will be needed to obtain more reliable evidence.

Endoscopy_UCTN_Code_TTT_1AO_2AG

## Notes

Endoscopy E-Videos https://eref.thieme.de/e-videos
*E-Videos* is an open access online section of the journal *Endoscopy* , reporting on interesting cases and new techniques in gastroenterological endoscopy. All papers include a high-quality video and are published with a Creative Commons CC-BY license. Endoscopy E-Videos qualify for HINARI discounts and waivers and eligibility is automatically checked during the submission process. We grant 100% waivers to articles whose corresponding authors are based in Group A countries and 50% waivers to those who are based in Group B countries as classified by Research4Life (see: https://www.research4life.org/access/eligibility/ ). This section has its own submission website at https://mc.manuscriptcentral.com/e-videos .
